# Should Deferred Stenting Still Be Considered in ST-Elevation Myocardial Infarction with High Thrombus Burden?

**DOI:** 10.3390/jcdd8060059

**Published:** 2021-05-21

**Authors:** Xenofon M. Sakellariou, Georgios I. Katsanos, Andreas P. Efstathopoulos, Dimitrios G. Sfairopoulos, Konstantinos V. Stamatis, Spyridon D. Pappas, Theofilos M. Kolettis, Dimitrios N. Nikas

**Affiliations:** 1st Cardiology Department, University Hospital of Ioannina, 45500 Ioannina, Greece; gkatsanos@hotmail.com (G.I.K.); efstathoa@gmail.com (A.P.E.); dimitrios.sfairopoulos@gmail.com (D.G.S.); kwstas.stamatis100@gmail.com (K.V.S.); spyrosdpappas@gmail.com (S.D.P.); theofilos.m.kolettis@gmail.com (T.M.K.); dimitrios.nikas@gmail.com (D.N.N.)

**Keywords:** acute myocardial infarction, primary percutaneous coronary intervention, high thrombus burden, micro-vascular obstruction, stent implantation, deferred-stenting

## Abstract

Patients with ST-elevation myocardial infarction (STEMI) treated with primary percutaneous coronary intervention (PCI) may demonstrate distal microvascular embolization of thrombotic materials. We retrospectively examined 20 cases displaying extensive thrombus in the infarct-related artery (IRA), treated either with a two-step procedure, with interim tirofiban infusion, or immediate stent implantation. Distal embolization tended to be more common in the latter strategy, but, overall, the outcome was comparable. Thus, a two-staged procedure may be considered in selected cases of primary PCI associated with high thrombus burden.

Primary PCI is the preferred reperfusion strategy in patients with STEMI; however, its value is compromised by distal micro-embolization, occurring in as many as 10% of cases of apparently successful procedures [1]. The extent of the angiographic thrombus has been identified as a powerful predictor of such events, which are mediated by vasoconstrictors and inflammatory cytokines released from neutrophil-platelet aggregations [2]. In the presence of a high thrombus burden, the approach of acutely restoring blood flow without stenting has been examined [3,4,5], although the results were conflicting. Deferred-stenting was disfavored after the results of a larger trial [6], which showed no effect on major clinical outcomes. Regardless, the debate resurfaced after the findings of three meta-analyses [7,8,9], indicating lower risk for myocardial injury by this approach.

To contribute to the ongoing discussions, we retrospectively examined cases of STEMI treated with primary PCI during the calendar year 2017, displaying extensive thrombus in the infarct-related artery. Of 34 such cases identified, 12 were excluded because of multi-vessel disease, and 2 because of concurrent anticoagulation. A two-stage procedure, consisting of acute restoration of blood flow in the absence of stenting, followed by tirofiban infusion and repeat angiogram after 3 days was followed in 10 cases. These patients, hereafter referred to as deferred-stenting group, were compared with the remaining 10 patients, referred to as immediate-stenting group. As shown in Table 1, the demographic, clinical, and angiographic characteristics were similar in the two groups. 

All angiograms showed totally occluded IRA, with adequate (TIMI ≥ 2) flow restored after dilatation with a small-diameter balloon. Aspiration thrombectomy as an adjunctive therapy was not used with any patient. All patients referred to significant relief of pain and there was more than 50% ST-segment resolution in the single lead showing maximum ST segment at baseline ECG. The presence of a high thrombus burden (defined as thrombus length ≥ 2× diameter) was confirmed after quantification by the TIMI scoring system. In the deferred-stenting group, the first PCI was followed by an initial bolus tirofiban administration of 25 μg/kg given over a 3-min period in the catheterization laboratory, followed by a continuous intravenous infusion (0.15 μg/kg/min for 24 h) and subcutaneous enoxaparin (1mg/kg every 12 h), based on the previously demonstrated efficacy of this regimen on thrombus burden [3]; repeat coronary angiography was performed after 72 ± 16 h.

We compared angiographic and clinical variables, as well as indices of myocardial necrosis between the two groups. Categorical variables were compared with chi-square, whereas normally distributed continuous variables (as per Kolmogorov–Smirnov) were compared with t-test; significance was set at *p* < 0.05.

No re-occlusion of the infarct-related artery or major bleeding was recorded during hospital stay. Distal embolization was noted in three patients after immediate-stenting, but was absent after deferred-stenting. However, myocardial blush grades, peak troponin levels, and final left ventricular ejection fraction did not differ between the two groups, as seen in Table 2.

Thrombus burden decreased markedly during the second PCI in the deferred-stenting group, whereas the stenosis severity was lower than the immediate-stenting group. In three patients in the deferred-stenting group displaying stenoses <50%, no stents were implanted during the procedure. Figure 1 shows a representative example from the deferred-stenting group.

In addition to its retrospective design, the major limitation of the study is the small number of patients; hence, the information provided here should be examined only in the context of previous reports [3,4,5,6,7,8,9], Under this prism, our results indicate that deferred-stenting during STEMI may be considered in the presence of high thrombus burden, provided that an adequate coronary flow is established by minimal intervention during the initial PCI. This approach may decrease distal embolization, albeit without apparent effect on final infarct size or overall left ventricular function. Short-term tirofiban infusion decreases thrombus burden and may obviate the need for stent implantation in some patients. We feel that the deferred-stenting approach is presented as an option in selected cases, deemed at high risk for microvascular obstruction, until further data are available from the ongoing PRIMACY trial [10].

## Figures and Tables

**Figure 1:**
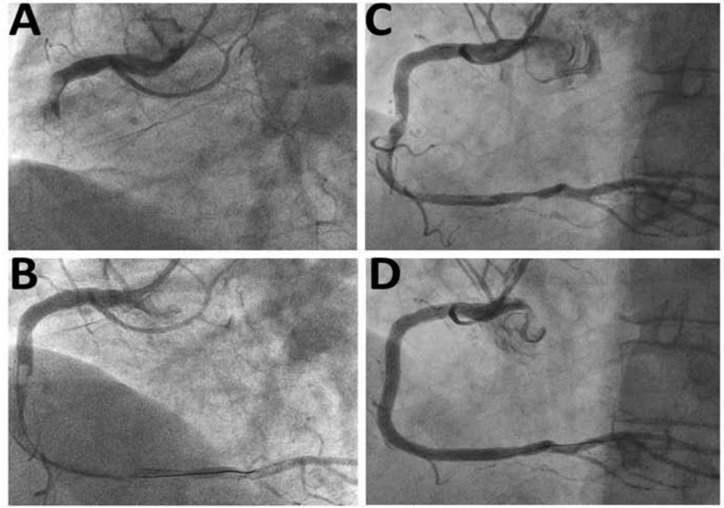
Representative case managed by deferred-stenting. (**A**) Presentation with total occlusion of the right coronary artery and high thrombus burden; (**B**) Restoration of satisfactory coronary flow after dilatation with 2.0 mm balloon. Note the extensive thrombus occupying large part of the right coronary artery; (**C**) Repeat angiogram after 48 h showing decreased thrombus burden; (**D**) Final result after stent implantation.

**Table 1:** Baseline characteristics (SD: standard deviation; NS: non significant; CABG: coronary artery bypass grafting; LAD: left anterior descending artery; LCx: left circumflex artery; RCA: right coronary artery; TIMI: thrombolysis in myocardial infarction).

		Immediate-Stenting (*n* = 10)	Deferred-Stenting (*n* = 10)	*p*
Demographics	Age [mean (SD)], years	68 ± 15	61 ± 12	NS
	Male sex	9	10	NS
Risk factors	Current smoking	5	5	NS
	Family history	2	3	NS
	Obesity	2	1	NS
	Diabetes mellitus	3	3	NS
	Hypertension	4	6	NS
	Dyslipidaemia	6	4	NS
History of coronary artery disease	Myocardial infarction	2	1	NS
	Previous PCI	2	1	NS
	CABG	1	1	NS
Culprit artery with thrombus	LAD	1	1	NS
	LCx	2	1	NS
	RCA	7	8	NS
Antithrombotic treatment	Aspirin + Clopidogrel	7	7	NS
	Aspirin + Ticagrelor	3	3	NS
Transfer from non-PCI hospital		6	8	NS
Ejection fraction on admission [mean (SD)], %		43.5 ± 4.7	47 ± 5.3	NS
Symptom to balloon time [mean (SD)], min		59 ± 21	67 ± 18	NS
TIMI 2 flow grade after balloon dilatation		4	6	NS
TIMI 3 flow grade after balloon dilatation		6	4	NS
Grade 4 thrombus burden after acute blood flow restoration		10	10	NS
Culprit artery stenosis after acute blood flow restoration [mean (SD)], %		92.9 ± 3.9	91.1 ± 5.8	NS
Myocardial blush grade after acute blood flow restoration		2.1 ± 0.57	2.3 ± 0.48	NS
Distal embolization after acute blood flow restoration		0	0	NS

**Table 2:** Outcome (SD: standard deviation; NS: non significant).

	Immediate Stenting (*n* = 10)	Deferred Stenting (*n* = 10)	*p*
Grade 4 thrombus burden before stent implantation	10	0	0.001
Culprit artery stenosis before stent implantation [mean (SD)], %	92.9 ± 3.9	77.5 ± 16.9	0.011
Myocardial blush grade	2.4 ± 0.52	2.7 ± 0.48	NS
Distal embolization after stent implantation	3	0	0.06
Need for stent implantation	10	7	0.06
Peak troponin concentration [mean (SD)], pg/mL	11,186 ± 9.836	10,005 ± 9.705	NS
Peak Creatinine [mean (SD)], mg/dl	1.35 ± 0.51	1.16 ± 0.28	NS
Pre-discharge ejection fraction [mean (SD)], %	50 ± 5.2	53.5 ± 3.4	NS

## Data Availability

The data presented in this study are available within the article.
